# Urinary Biomarkers of Mycotoxin Induced Nephrotoxicity—Current Status and Expected Future Trends

**DOI:** 10.3390/toxins13120848

**Published:** 2021-11-28

**Authors:** Zsolt Ráduly, Robert G. Price, Mark E. C. Dockrell, László Csernoch, István Pócsi

**Affiliations:** 1 Doctoral School of Molecular Medicine, University of Debrecen, H-4032 Debrecen, Hungary; 2 Department of Physiology, Faculty of Medicine, University of Debrecen, H-4032 Debrecen, Hungary; csl@edu.unideb.hu; 3 Department of Nutrition, Franklin-Wilkins Building, King’s College, 150 Stamford St., London SE1 9NH, UK; robert.price@kcl.ac.uk; 4 South West Thames Institute for Renal Research, Carshalton, Surrey SM5 1AA, UK; mark.dockrell@nhs.net; 5 Department of Molecular Biotechnology and Microbiology, Faculty of Science and Technology, University of Debrecen, H-4032 Debrecen, Hungary; pocsi.istvan@science.unideb.hu

**Keywords:** nephrotoxicity, mycotoxin, biomarkers, AKI, NAG, KIM-1, ochratoxin A, citrinin, NGAL

## Abstract

The intensifying world-wide spread of mycotoxigenic fungal species has increased the possibility of mycotoxin contamination in animal feed and the human food chain. Growing evidence shows the deleterious toxicological effects of mycotoxins from infants to adults, while large population-based screening programs are often missing to identify affected individuals. The kidney functions as the major excretory system, which makes it particularly vulnerable to nephrotoxic injury. However, few studies have attempted to screen for kidney injury biomarkers in large, mycotoxin-exposed populations. As a result, there is an urgent need to screen them with sensitive biomarkers for potential nephrotoxicity. Although a plethora of biomarkers have been tested to estimate the harmful effects of a wide spectrum of toxicants, β_2_-microglobulin (β_2_-MG) and *N*-acetyl-β-D-glucosaminidase (NAG) are currently the dominant biomarkers employed routinely in environmental toxicology research. Nevertheless, kidney injury molecule 1 (KIM-1) and neutrophil gelatinase-associated lipocalin (NGAL) are also emerging as useful and informative markers to reveal mycotoxin induced nephrotoxicity. In this opinion article we consider the nephrotoxic effects of mycotoxins, the biomarkers available to detect and quantify the kidney injuries caused by them, and to recommend biomarkers to screen mycotoxin-exposed populations for renal damage.

## 1. Introduction

The kidney is a multifunctional organ, the structural and functional unit of which is the nephron. The nephron consists of a variety of different cells which perform highly complex and precisely orchestrated physiological processes. Any stimuli which disturb these cells, and their interplay may induce kidney damage and subsequent endocrine dysfunction, cardiovascular disease, metabolic disorders, and further renal failure. The kidney is particularly susceptible to nephrotoxic attack, owing to its high blood flow and specialized metabolism. Environmental pollutants which target the kidney include metals, solvents and naturally occurring compounds, including aristolochic acid and mycotoxins [1]. Fungi or molds grow on a variety of foodstuffs including cereals, nuts, and fruit. Mycotoxins are naturally occurring toxic compounds produced by a wide spectrum of different microfungi. More than 500 mycotoxins have been identified, but few of them normally occur in the human diet in significant amounts and, consequently, affect human and animal health [2,3]. Oral exposure via contaminated food or feed is considered the predominant exposure route and represents a serious health issue. Low or moderate mycotoxin exposure over years can result in large economic loss and severe health effects in animals and man [4].

**Figure 1:**
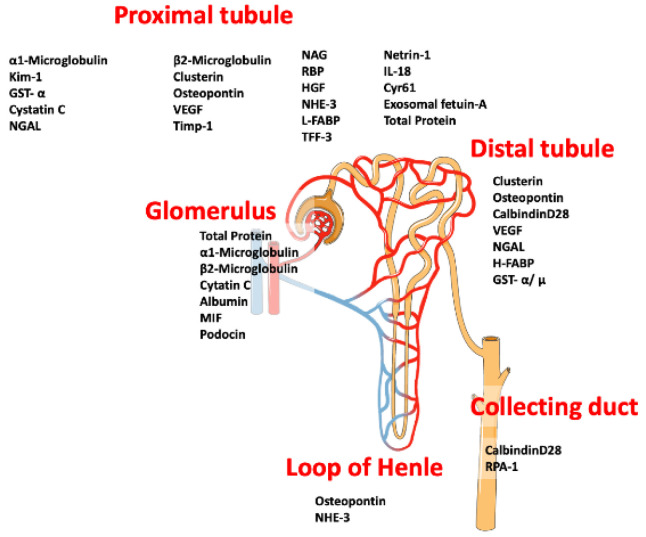
Biomarkers used to assess renal damage on the different sections of the nephron. Several putative urinary biomarkers are shown. Some of them are under study while other are well established. Modified from [8].

As the majority of transport processes involving nephrotoxic compounds or their derivatives occurs via the renal glomeruli and the proximal tubules, these are the main targets for xenobiotics, including mycotoxins [1,5]. Significant efforts have gone into studying those biomarkers, which could indicate the locus of the affected cells, furthermore, microRNA in the urine, assessment of exosomes and other extracellular vesicles are in focus as well [1]. As an example, microfungi and heavy metals can induce renal cell apoptosis, where both intrinsic and extrinsic cell death signaling pathways are involved. Much of the available experimental data has not yet been translated into robust accepted in vitro or ex vivo models to study nephrotoxicity in man. Furthermore, the growing body of evidence on the versatile nephrotoxic effects of various fungal secondary metabolites warns us that we still have a long way to go in the development and implementation of suitable diagnostic and therapeutic approaches with which to screen for the deleterious effects of these compounds [1,5]. To estimate the renal damages caused by nephrotoxic mycotoxins, a wide array of urinary biomarkers should be tested (Figure 1). In mycotoxin research field “biomarker” refers to the direct measurement of the excreted mycotoxin or a breakdown product [6]. This provides information on the degree of exposure, but no information on the adverse cellular or organ effects. To date, few studies have used urinary enzymes or kidney injury biomarkers to monitor the nephrotic effect of a mycotoxin. An early study of the effect of ochratoxin A in rats is promising but equivalent studies in humans are still lacking [7].

## 2. Mycotoxin Exposure

As a result of world-wide climate changes, mycotoxigenic microscopic fungi are spreading rapidly, imposing serious health risks on both household animals and consumers causing major economic losses [9,10,11,12]. The major mycotoxin producer microfungi belong to the genera *Aspergillus*, *Fusarium* and *Penicillium*. Their highly toxic secondary metabolites have carcinogenic, genotoxic, teratogenic, neurotoxic, hepatotoxic, nephrotoxic as well as with immune and endocrine systems disturbing properties [9,10,11,12]. Although mycotoxin contaminants typically represent a major threat to populations living in tropical regions [12,13], the boundaries of high-risk zones is moving north in the northern hemisphere [12]. Mycotoxins represent one of the major environmental toxicants threatening human health today. Exposure can be via food consumption, inhalation, dermal contact or through occupational exposure [6,14,15]. The most prevalent toxins in the food chain are: aflatoxins (AFs), ochratoxin A (OTA), patulin (PAT), fumonisins, citrinin (CIT), ergot alkaloids, trichothecenes like deoxynivalenol (DON), T-2 toxin (T-2) and zearalenone (ZEN) [16]. Because of the growing concern about mycotoxin-associated health problems including kidney injury, a search for suitable markers to quantify mycotoxin toxicity is urgently required. The most predominant exposure is to OTA and CIT, current methods for detecting exposure include just the determination of urinary levels of mycotoxins and/or their excreted forms [6,17,18,19]. As mycotoxins are naturally occurring secondary metabolites are often consumed by people, the exact nephrotoxic mechanisms should be clarified as well.

Ochratoxins are produced by *Aspergillus ochraceus*, *Aspergillus carbonarius*, and *Aspergillus niger*, which have a world-wide distribution and OTA can be found in a wide variety of agricultural products, including cereals, grapes, oil seeds, wine, and barley. According to the WHO guidance, the PTWI (provisional tolerable weekly intake) of OTA is 112 ng/kg body weight (bw) [20]. OTA has been classified by the International Agency for Research on Cancer (IARC) as a group 2B human carcinogen [19,21,22]. After biotransformation in the human body, more than 20 OTA derivatives have been described to date. OTA forms covalent DNA adducts through free radical and benzoquinone intermediates, moreover the OTA hydroquinone (OTHQ) metabolite can undergo an autoxidative process to generate the quinone electrophile OTA quinone (OTQ) that also reacts with DNA. Therefore, the formation of OTQ or phenoxy and aryl radicals can result in increased Reactive Oxygen Species (ROS) production that is mainly responsible for its cytotoxicity [12]. Possible mechanisms leading to OTA nephrotoxicity as well as its known hepatotoxicity and immunotoxicity may be linked to the inhibition of protein synthesis and lipoperoxidation. The modulation of the MAP kinase cascade has been reported in some cases as well [20,23,24]. OTA binds strongly to albumin, so its elimination by glomerular filtration is negligible. Instead, tubular secretion is the main excretion mechanism in kidney, and the tubular reabsorption could be partially responsible for intracellular accumulation of OTA [25]. Organic anion transporters (OATs) are membrane transport proteins and can play a role in transport processes of OTA. The basolateral OATs (OAT1 and OAT3) are mainly responsible for the uptake from blood into the tubular kidney cells, the apical OAT4 transporter may have a role in urinary reabsorption of OTA, resulting in its accumulation in the kidney [26].

As CIT often co-occurs with OTA and they are often analyzed together. According to the classification of IARC, CIT is a group 3 human carcinogen and is not classified in terms of its carcinogenicity in humans [21,27]. CIT has a “level of no concern for nephrotoxicity” (a provisional tolerable daily intake—“PTDI”) value of 0.2 μg/kg bw. It is produced by several species in the genera *Monascus, Aspergillus* and *Penicillium*, and it occurs principally in plant products and stored grains [28,29]. CIT and OTA have also been associated with alterations in renal function and/or with the development of renal pathologies [30]. However, because of the shortage of available data, several questions have emerged, as outlined in the Balkan Endemic Nephropathy (BEN) report. Although the exact toxicity of CIT are not fully understood, CIT might exerts its toxic effect by altering mitochondrial function and it Ca^2+^ homeostasis [28]. It has also been reported that CIT treatment resulted in swollen and degenerated mitochondria in renal cortical cells of pigs, broiler chickens and laying hens as well [31]. Furthermore, it interferes with the electron transport chain, which results in oxidative stress, CIT also interferes with cholesterol and triglyceride metabolism. Acute intoxication is rare and appears mainly in feed, a small amount of data is available with the adverse effects of long term CIT intake [29]. While CIT has been reported as a non-mutagenic mycotoxin, some authors have pointed out that it can cause chromosomal abnormalities in bone marrow cells of mammals [28]. Co-exposure to CIT and OTA simultaneously modifies DNA adduct formation with increasing appearance of the C-C8dG-OTA adduct [32]. Recently, several studies have been carried out with other mycotoxins as well. Kidney cell exposure to ZEN increased caspase-3 activity, malondialdehyde (MDA, a lipid peroxidation product) concentrations. IL-10, IL-6, TNF-alpha, Bax mRNA levels are decreased together with total antioxidant activity and down-regulated expression of *GSH-Px*, *CAT* and *BCL-2* mRNA [33].

PAT is a water soluble, colorless, polyketide lactone, which is thought to exert its toxicity through reacting with thiol groups (cysteine, glutathione, thiol moieties of proteins) in the cytoplasm [34]. A recent study demonstrated p53 activation contributes to patulin-induced nephrotoxicity via modulation of ROS generation [35].

Fumonisins are structurally similar to cellular sphingolipids and they have been shown to inhibit sphingolipid biosynthesis at ceramide synthase [36]. The primary amino and tricarballylic acid groups of the toxin are responsible for the reaction with ceramide synthase. Fumonisin-induced toxicity often results in apoptosis, alteration in cytokine expression or generation of oxidative stress [37]. IARC has classified fumonisin B1 (FB1) in toxicity group 2B as probably carcinogenic [21].

Although the acute oral toxicity of sterigmatocystin (STC) is not well described, the main target organs in humans are the kidney and liver [17]. At doses between 10 and 100 mg/kg body weight, STC causes hyaline casts in the collecting tubules, pyknotic nuclei in the tubular cells at the cortico-medullary junctions as well as cortical hemorrhage. Hyaline degeneration or necrosis of tubular epithelial cells were detectable as well. At higher doses in the range of 100–144 mg/kg body weight, lesions were accompanied by degeneration and necrosis of glomeruli with hyaline thickening of the basement membrane. Massive hemorrhage and necrosis are also seen when the dose was greater 144 mg STC/kg body weight [38].

More than 20 types of aflatoxins (AFs) have been identified to date, and of these AFB1, AFB2, AFG1 and AFG2 have proved to be the most toxic to both humans and livestock. As a consequence, IARC has classified these mycotoxins as group 1 carcinogen, which means that they are carcinogenic to humans [39,40,41]. The consumption of high amount of AFB1 leads to aflatoxicosis, which is a serious and emerging problem in the world. Although AF-related notifications in food and feed, based on the European Union (EU) Rapid Alert System for Food and Feed (RASFF) is working well, similar methods should be introduced in developing countries as well [12,41,42]. Oxidative damage in a tissue is often caused by AFs, when ROS, e.g., hydrogen peroxide, hydroxyl radicals, superoxide radicals are generated. Their levels are often exceed the antioxidant capacity of the cells or they decrease the level of the defense mechanisms. Epidemiological surveys and studies have described various kidney and liver disorders within AFs- affected populations [42]. As AFs are among the most dangerous and best studied mycotoxins, a considerable amount of literature and experimental data are available, including urinary and fecal excretions of different AF metabolites [43,44]. 

The mycotoxins discussed above are listed in Table 1. The nephron segments affected, and doses studies are summarized. For brevity, Table 1 contains only a selection of the relevant literature to demonstrate the connection between nephrotoxicity and mycotoxins.

The primary source of mycotoxin exposures for humans is dietary intake and, importantly, foods typically contain a versatile and hardly predictable mixture of mycotoxins rather than a sole mycotoxin contaminant [27,48,49]. There are three different ways for mixed mycotoxin contaminations to occur in food. Firstly, many fungal species are capable of producing a wide spectrum of different toxins, secondly food can be contaminated by more than one type of mycotoxin producer microfungi and thirdly diets are mixtures and could contain several different mycotoxin contaminants. Each of these routes can be harmful for the consumer. All of these mycotoxins are typically metabolized in the liver and are excreted via the kidneys, causing severe injuries and abnormalities in these organs [5,17,20,28,50].

Although CIT and OTA are rarely produced by the same species, *Penicillium verrucosum* Dierckx is actually a good exception, which does produce both OTA and CIT, which considerably contributes to the mycotoxin content of cereals in cool temperature climates. Nevertheless, OTA is the main toxin and CIT is produced in smaller quantities [51]. Furthermore, an *A. niger* strain was isolated from maize in Portugal, which was able to produce fumonisins and OTA at the same time [51]. As a consequence of global trading and transportation of goods, cereals, grapes, fruits and nuts are susceptible to contamination [51,52,53] and, because human diet is a complex mixture of various ingredients, multiple mycotoxicosis is a rising problem world-wide [48,54]. Although the most affected regions are still in low GDP Third World countries [11,12], the mycotoxigenic fungal species preferring warm climates are currently moving northwards [12], a result of climate change. Not surprisingly, co-occurrence of mycotoxins in food and animal feed is a severe problem not just in Africa, but also in Europe [55]. 

As mentioned above, CIT is mainly produced by the genera *Penicillium* and *Monascus*, while the genera *Pencillium* and *Aspergillus* are the main OTA producers [27,28]. Concomitantly, increased OTA and CIT concentrations in food can increase the prevalence of tubule-interstitial nephropathy [27]. A study on HK-2 cells (human proximal tubule-derived epithelial cells) revealed, that physiological relevant OTA and CIT can act synergistically, furthermore prolonged exposure could result in chronic kidney disease and tubulointerstitial fibrosis. These features can cause complete renal failure, particularly in vulnerable individuals [27,51].

Although several investigations have been carried out to describe and explain the toxicological events elicited by combined mycotoxin exposures [27,56,57], and the number of on-going research projects is rapidly increasing in this field, it would be basically important now to define new safe intake limits for mycotoxin combinations. Furthermore, it would be useful to monitor the physiological conditions including renal functions of people exposed to mycotoxin mixtures [12].

It is encouraging that a recent study in UK of children by Gratz et al. (2020) investigated multi-mycotoxin exposure and found that the principal source of mycotoxins was cereal foods. As breakfast cereal is liked and often consumed by children, their mycotoxin exposure is even higher than the more resistant adults. In this study, spot urine samples were analyzed for multi-mycotoxins using liquid chromatography-coupled tandem mass spectrometry [58]. 

Mycotoxicosis can occur at every stages of life in both humans and livestock, although the earlier the exposure, the more severe the long-lasting toxicological consequences [12,31,59,60]. As a result, WHO has recommended lower PMTDI limits in baby and child food, while in EU countries these limits are under strict control as well [61,62]. Importantly, adverse pregnancy outcomes, like abnormally lower body weight, neonatal jaundice, eclampsia, small head size or stillbirth may occur frequently in cases where high-level maternal mycotoxin exposure had occurred [63]. Although the complexity of placenta can protect the embryo and the fetus during intrauterine life, the mycotoxin exposure may contribute to several developmental disorders, even affecting the kidneys A recent study revealed that pregnant women are often exposed to combined effects of OTA and CIT [56], which may affect kidney development as well. In utero renal development in man continues up to birth with peak growth during the third trimester. Consequently, preterm and low birthweight infants tend to have a reduced nephron number [64]. Low nephron number, in turn, results in a significantly greater vulnerability to acute and chronic kidney damage [65]. 

After birth, infants are mainly dependent on their mothers in terms of nutrition and the most suitable nutrient for them is breast milk. Breastfeeding is more critical in those countries where the infant formulas are not available [66]. Breast milk provides developmental and psychological advantages to infants, although it could be even harmful if toxic agents, like mycotoxins, are secreted into breastmilk [66,67]. Interestingly, the colostrum may contain more OTA than the mature milk, so the severity of OTA exposures in infants could even be worse than those found for 4–6-month-old children [68]. 

The deleterious effects of mycotoxin exposure on renal functions of infants and children should therefore be investigated from as early as the neonatal period and should be considered separately from adults [69]. The occurrence of AKI may be frequent in even young age cohorts gives rise to two questions: firstly—how does mycotoxin toxicity affect the health of exposed patients and how best can any pathological changes in renal function be detected and quantified [70,71,72,73].

## 3. Currently Available Methods to Detect Nephrotoxic Effects of Mycotoxins

Biomonitoring studies have provided us with a better understanding of the extent of mycotoxin exposures in an affected population [74]. Recent research has focused mainly on the metabolites which are produced via biotransformation after mycotoxin consumption [20,73,74]. Typically blood or urine samples are analyzed and different analytical techniques have been developed to quantify and detect individual mycotoxins and their derivatives [9,19]. It is noteworthy, that there is an increase in studies dealing with breast milk as a biological matrix to information about the carry over rate of mycotoxins from food [60,66,73,75]. Although the available human toxicokinetic data is constantly increasing, the concentration of a metabolite is difficult to relate to clinical or pathological changes in affected individuals. Currently, studies are focusing on sensitive groups, e.g., pregnant women, infants or children, to avoid mycotoxicosis as early as possible [56,60,63,67,76,77]. 

Although it is really difficult to monitor the exact consumption of mycotoxin-contaminated food [78,79], a seasonal and regional difference in OTA exposure was found in a study carried out in Bangladesh [78]. While OTA is found worldwide, it is a particular problem in Mediterranean countries, including Italy, Spain, and Greece [80,81,82,83], as well as several African countries, e.g., Cameroon, Senegal, Benin, and Nigeria [84,85]. In order to assess OTA exposure, urine and blood are analyzed together with its intake via food and from air. Interpretation of OTA exposure is currently difficult due to the lack of specific and validated biomarkers and detailed toxicokinetic. 

CIT occurs mainly in plant products and stored grains as a consequence of *Penicillium* and *Aspergillus* infestation. Unfortunately, *Monascus* fermentation products, like red mold rice, used as food supplements, may also contain CIT. Recently, a new EC Commission Regulation has been published (EU 2019/1901) amending Regulation (EC) No 1881/2006. The maximum level of CIT in food supplements is lowered from 2000 μg/kg to 100 μg/kg, based on rice fermented with “red yeast” *Monascus purpureus* [86]. Biomonitoring studies have been carried out in Germany, the Czech Republic, and Bangladesh; however, the results could be improved if larger CIT affected areas were included [87]. The appearance of CIT and its metabolite, dihydro-citrinone (DH-CIT) has frequently been monitored in urine, however, the correlation with renal failure has not been established yet. Recent publications have reported, that the co-occurrence of mycotoxins (e.g., FB1, CIT, ZEN) can be more dangerous to health than the exposure to a single mycotoxin [51,88,89,90]. It is noteworthy that both cell culture and histological kidney model systems are currently available to test the renal toxicity of environmental toxicants, including mycotoxins. Hence, it is relatively easy to test for any deleterious effects and propose mechanisms of actions in vivo [91]. Although tracking mycotoxins and metabolites in body fluids and excreta could give a good picture about their intake, transformation, and secretion, less is known of their toxicological effect in vivo. In order to clarify the nephrotoxic effects of different mycotoxins and their derivatives in vivo, the implementation of novel urinary biomarkers in in vitro studies seems to be unavoidable [8,18,92].

## 4. Novel Biomarkers for the In Vivo Nephrotoxic Effects of Mycotoxins

**Figure 2:**
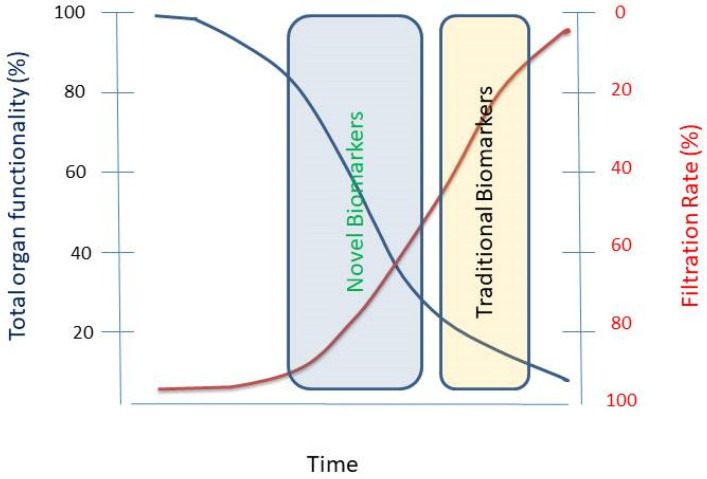
Schematic representation of sensitivity of traditional and novel biomarkers of nephrotoxicity. Filtration Rate (red line), the amount of blood filtered through the glomerulus per minute, is the clinical measure renal function (blue line). Unfortunately, the most widely accepted indicators of filtration rate lack sensitivity. Novel biomarkers have the potential of detecting damage before it becomes irreversible. Modified from [8].

As the incidence of acute kidney injury (AKI) and chronic kidney disease (CKD) is increasing worldwide, early identification and prompt intervention are needed to preserve the health of the patients. Traditional renal biomarkers, including estimated glomerular filtration rate (eGFR), blood urea nitrogen (BUN) and serum creatinine (sCr) have been used to detect kidney failure, however, these markers are insensitive and rather non-specific. In order to detect early-stage renal injury, recent research has started to concentrate on more sensitive procedures than eGFR, BUN and sCr (see Figure 1 and Figure 2). Using traditional methods to estimate kidney function, e.g., sCr in neonates and children, make clinical decisions difficult, because of variability in different stages of AKI [72,93]. Recently, new biomarkers have been described which reflect changes in the kidney at an early stage. In addition, genomic and proteomic technologies are also now available. Urinary level of novel biomarkers, like cystatin C, NGAL may indicate the severity of AKI in children [72]. 

Novel renal biomarkers are defined as parameters of physiological, chemical, structural, and genetic changes that show the severity, progress or presence of a histopathological alteration [94,95]. As an example, the Food and Drug Administration (FDA) and the European Medicines Agency have qualified KIM-1 to monitor the safety of drug induced nephrotoxicity [95]. We briefly introduce some molecules as potential biomarkers to monitor the toxic effects of mycotoxins on kidney.

β_2_-microglobulin (β_2_-MG) is a component of MHC class I molecules, and these protein complexes also incorporate α_1_, α_2_, and α_3_ proteins which are present on the surface of all nucleated cells. It is encoded by the *B2M* (human) and *B2m* (rat) genes and it has no transmembrane region, furthermore, β_2_-MG associates with other molecules, like CD-1 or MR-1 [96]. This biomarker can be used to distinguish glomerular and tubular disorders in kidney, owing to the passage of β_2_-MG through the glomeruli and their consecutive reabsorption by the renal proximal tubules. Under normal conditions, just minute quantities of β_2_-MG are excreted in the urine, however, β_2_-MG concentrations will increase when the renal tubules become damaged or diseased and, consequently, the efficiency of reabsorption becomes very low. On the contrary, when the glomeruli are damaged they are unable to filter out β_2_-MG, so the level of β_2_-MG in the blood rises [8,96]. β_2_-MG is still frequently used e.g., in heavy metal nephrotoxicity screening programs, although this biomarker is being replaced by other, more reliable markers, because of its instability in urinary samples at pH < 7 and its possible partial degradation before analysis [97].

*N*-acetyl-β-D-glucosaminidase (NAG) excretion is a sensitive indicator of proximal tubule injuries. It can be found in the lysosomes of proximal tubule epithelial cells. As it has a high molecular mass, 130 kDa, no NAG filtration occurs in the glomeruli, instead, it is released into the urine from dying or misfunctioning brush border cells as a result of tubular injury [98]. Nowadays, NAG is one of the most widely used biomarkers to diagnose renal tubular injury [98], and it is used routinely as a reference test in assessing renal tubular injuries in both human and in vivo animal nephrotoxicity models [99,100].

Kidney injury molecule-1 (KIM-1) is a type 1 transmembrane glycoprotein encoded by the *HAVCR1* (human) and *Havcr1* (rat) genes. KIM-1a and KIM-1b are the two homologs in human, however, only KIM-1b is expressed in kidney orientated cells [94]. In a normal kidney, the expression level is relatively low, however, after renal tubular injury an increased expression can be detected. S3 segment of the proximal tubule is the locus where KIM-1 expression and synthesis reach the highest level [101]. After tubular injury, the membrane bound segment of KIM-1 is released in a metalloproteinase-dependent process, and the 90 kDa peptide segment can be detected in the urine. As the expression of KIM-1 correlates with interstitial fibrosis and inflammation progression in the affected kidneys, making KIM-1 a sensitive biomarker [102]. The ectodomain is soluble in urine and is stable at room temperature as well, which provides KIM-1 based assays with good reliability and reproducibility [94]. 

Neutrophil gelatinase associated lipocalin (NGAL or siderocalin, lipocalin-2) is encoded by the *LCN2* and *Lcn2* (human and rat, respectively) genes and its expression can be detected in the loop of Henle and the collecting ducts. It protects the tubular epithelial cells from cell death by upregulating heme oxygenase-1 [102,103]. NGAL is among the top upregulated genes in damaged kidneys and a promising biomarker of tubular damage because this protein enters both serum and urine rapidly after the onset of AKI [104]. It can pass freely through the glomeruli as its molecular weight is 25 kDa, it is stable and resistant to protease degradation. Following filtration NGAL is retrieved by the proximal tubular cells by endocytosis [104]. 

Some other biomarker candidates have also been tested in recently [105,106], including monocyte chemoattractant protein-1 (MCP-1), which is mainly produced by tubular epithelial cells, and is a member of the C-C chemokine family. Among other potential applications, MCP-1 is considered to be a potential predictor of the degree of kidney fibrosis in IgA nephropathy patients [94]. Increased expression of the tissue inhibitor of metalloproteinases-1 (TIMP-1) may be indicative of the interstitial fibrosis of the kidneys [102].

In vitro nephrotoxicity studies using various kidney cell cultures, kidney models and in vivo studies with animal models are critically important to identify and validate novel renal injury biomarkers for human screening programs [27,107]. Nephrotoxicity studies carried out with mycotoxins have focused on OTA to date. As an example, the up-regulation of Kim-1, Timp-1, lipocalin 2, osteopontin, clusterin, vimentin and cyclooxygenase 2 genes have been reported in the kidneys of rats after long-term OTA exposures, potentiating the selection of future biomarkers to monitor OTA induced kidney injuries [7,108,109]. Increased urinary KIM-1 levels have been shown to correlate with subtle histopathological alterations in the kidneys in high OTA dose animal experiments [106]. The same study also reported elevated urinary β_2_-MG, cystatin C and calbindin excretions in rat.

Understanding the nature and extent of FB1 nephrotoxicity is hampered by the paucity of human clinical and epidemiological data. Combining the study of established markers of FB1 exposure with nephrotoxicity biomarkers would be extremely useful in establishing the extent of the problem. Exposure to FB1 decreased cell viability, induced apoptosis and concomitantly upregulated the expression of KIM-1, collagen I, α-SMA and TGF-β_1_ in transformed human HK-2 cell cultures [110]. In addition, autophagy was activated after FB1 exposure, including the conversion of LC3 and up-regulation of ATGs, and the autophagy inhibitor 3-MA could block FB1-induced abnormalities. Antioxidant enzymes (Gpx1 and Gpx4) were downregulated, and intracellular ROS levels increased when the concentration of FB1 increased. FB1 induced autophagy in HK-2 cells [110], via autophagy mediated by mTORC1 instead of mTORC2 [110]. The expression of KIM-1 by HK-2 cells suggests that they are derived from the s3 proximal region of the nephron. 

As mentioned before, in studies involving monitoring mycotoxin exposure and its consequences, focus has principally been on the metabolites and breakdown products of mycotoxins [6,45,78,111,112]. A mycotoxin “biomarker” basically means the quantification of mycotoxin molecules and their derivatives in blood, urine or breast milk [29]. For example a recent study in Bangladesh [112], CIT metabolites were checked but no kidney injury biomarkers, such as KIM-1, NAG, NGAL or β_2_-MG were analyzed. A review in 2017 summarized the data available on different combinations of mycotoxins in biological matrices. In addition, studies focusing on OTA and its metabolites in the tested matrices, neither maternal nor infant urinary samples were checked for kidney biomarkers [66]. Furthermore, now it is a general protocol to take urine samples from the patients to detect mycotoxin exposure, but no indices of renal function are analyzed [88]. Since clinical laboratories facilities and methods are improving even in low GDP countries, introducing new, easy-to-use and affordable biomarker procedures would be very beneficial [13,56,113].

## 5. Biomarker Platform for Assay of Nephrotoxic Mycotoxins

Mycotoxins may cause both acute and chronic disease, and the majority of mycotoxicoses will affect kidney function as well [19,20,22,25,37,114]. The most appropriate urinary biomarkers should be introduced into routine clinical laboratory protocols allowing estimation of the extent and localization of the renal injuries caused by mycotoxins. The characteristics of an ideal mycotoxin kidney injury biomarker should be as described below [102,115,116]. The assay should be noninvasive, sensitive, easy-to-perform and affordable for a routine laboratory, with easy access to samples, including urine and blood. The toxicological pathways behind the mycotoxin-triggered release of the candidate biomarkers should be clarified and related to the biomarker to increase the specificity of the assays [90,117]. Biomarker selection should follow the determination of whether it is most relevant for monitoring mycotoxin-induced AKI or CKD [8,115,116]. The latter would be especially important when the long-lasting, adverse physiological effects of chronic mycotoxin exposures are estimated. The selected biomarkers should provide the clinician with valuable information supplementing the routine information of mycotoxin metabolite tests and standard clinical laboratory assays currently available [105]. Monitoring mycotoxin intake and their adverse effect on renal functions would be particularly important for pregnant, breastfeeding mothers and their children [59,66,68]. Because of the accumulating evidence of mycotoxin carry-over from livestock to consumers, mycotoxin exposure of household animals should also be considered [30,118,119]. Biomarker assays could be incorporated into Good Feeding Practice to filter out those animals, which have been exposed to mycotoxin contaminated feed above tolerance limits.

The most widely used biomarkers in other fields are KIM1, NAG, NGAL and β_2_-MG [120,121]. KIM-1 is a suitable marker for nephrotoxicity testing of drugs, and nowadays there is an increasing demand for KIM-1-based assays [101]. KIM-1 is generally measured by enzyme-linked immunosorbent assay (ELISA) or microparticle Luminex xMAP Technology assay [106]. Urinary NAG activity has been quantified either spectrofluorimetrically or spectrophotometrically using a wide-selection of substrates [71]. In order to measure NGAL, ELISA or a chemiluminescent microparticle immunoassay (CMIA) are available [106]. All these widely-used biomarkers indicate injuries at different segments of the proximal tubule (Figure 1) [102]. In a study on the effect of OTA in rats, possible early biomarkers were studied. The investigators singled out KIM-1 expression as putative marker of choice. In Table 2 we expand on this idea. The utility of biomarkers can be limited if there are not suitable methods for their measurement readily available. Hence, we include the common analytical tools for the markers suggested [7]. Consequently, a wide array of kidney parameters, including kidney biomarkers should be analyzed in parallel with mycotoxin metabolites [7,60,112,122,123]. A developed rapid test for the quantification of mycotoxins in urine could possibly combined with specific kidney injury tests would be a potential solution to test mycotoxin caused nephrotoxicity. Urinary NAG currently is the predominant enzyme assay used in industrial population screening programs and is well suited to screening in especially in low GDP countries. Use of fast KIM-1 tests including lateral-flow strip tests is particularly promising in developing countries [120,121]. 

Mycotoxin exposure in the young may result in serious health problems and, hence, screening of neonates, infants and children for their renal function should be considered [60,124]. As the elevation of traditional biomarker levels, like sCr values, is typically indicative of renal insufficiencies, diagnosis of mycotoxin-elicited kidney injuries in neonates and infants seems to be difficult without the introduction of novel biomarkers [72]. Lowering mycotoxin exposure during intrauterine life could prevent premature birth and, as a consequence, would decrease the risk of the manifestation of AKI in neonates [69,125]. We suggest that a novel biomarker platform incorporating at some of the available NAG, NGAL, KIM-1, Cystatin C and/or TIMP-1 assays would help in the assessment of the progression of AKI in children [126]. Currently used laboratory procedures to screen for tubular damage resulting from mycotoxin damage are listed in Table 2. As there is a low amount of available data, the “Mycotoxins” column based on two findings: available publications, where biomarkers have been used so far or the affected kidney regions by the mycotoxin have been specified so far.

**Table 2:** Laboratory based assays that could be used to screen for renal tubular damage in affected populations.

Biomarker	Mycotoxins	Technique(s)	References
NGAL	OTA, AFB1, CIT	Immunoassays ELISA	[7,102,127]
KIM-1	OTA, AFB1, CIT	Immunoassays (ELISA, MSD-ECL)	[7,127,128,129]
NAG	OTA, CIT	Enzymatic assays	[7]
Cystatin C	OTA, AFB1, CIT	Immunoassays, ELISA	[7,105,127,129]
L-FABP	OTA, CIT	Immunoassays, ELISA	[7]
β_2_-MG	OTA, CIT	Immunoassays, ELISA	[7]
TIMP-1	OTA	Immunoassays, ELISA	[7,129]
clusterin	OTA	Immunoassays, ELISA	[7]
osteopontin	OTA	Immunoassays, ELISA	[7]

## 6. Concluding Remarks

There is clearly a great potential benefit in detecting mycotoxin induced renal damage as early as possible and identifying the specific toxin involved. Combining detection of biomarkers of exposure with biomarkers of renal effects has the potential to prevent long term kidney damage and save lives. In spite of this, there are only very few recent publications involving urinary enzymes and mycotoxins together. Since the principal route of exposure is via contaminated food, it is important that the tests should be low cost and easily adapted to screen large populations. Kidney injury biomarkers are used to screen and monitor large populations exposed to nephrotoxins as well as to characterize the toxicological mechanism underlying their effect on the kidney. Screening programs should have both health and economic importance. Detection of mycotoxin-induced kidney injury at an early stage can allow intervention, thereby improving the health and the lives of people worldwide as well as reducing hospital costs. One of the objectives of the screening programs would be to develop easy to use point of care tests which would reduce the use of laboratory time and facilities. In addition, they would be suitable for use in developing countries which lack laboratory facilities. The established technology used in point of care testing could be utilized for screening. It should also be emphasized that certain mycotoxigenic microfungi, like the Aspergilli, are constantly moving north, which is still at relatively modest rate, but accelerating in the temperate zone in the northern hemisphere. It is time therefore to test urinary enzymes biomarkers to monitor mycotoxin-elicited renal damage in both household animals and humans. A combination of easy-to-use enzyme tests, e.g., NAG and KIM-1 with the direct assay of mycotoxins and their derivatives in urine would provide the best approach with which to determine both the effect and extent of exposure to mycotoxins and the detection of any nephrotoxic effects. 

## Figures and Tables

**Table 1:** Mycotoxins with established nephrotoxic effects.

Mycotoxin	Model System	Doses	Nephron Segment Involved	References
Ochratoxin A	mice, monkey, human	over 10 ng/kg bw	proximal tubules	[45]
Citrinin	mice, rabbit	35–200 mg/kg bw	proximal tubules	[28]
Zearalenone	mice	40 mg/kg bw	general kidney damage, not characterized	[33]
Fumonisin B1	quail chicks	200 ppm	proximal tubules, proximal convoluted tubules	[46]
Sterigmatocystin	mice, monkey	10–144 mg/kg bw	collecting ducts	[38]
Aflatoxin B1	mice	30–200 μg/kg bw	proximal tubule and general kidney damage	[42,47]

## Data Availability

No new data were created or analyzed in this study. Data sharing is not applicable to this article.

## Abbreviations

AF	Aflatoxin
AKI	Acute Kidney Injury
ATGs	Autophagy Regulated Genes
BUN	Blood Urea Nitrogen
*CAT*	Catalase gene (human)
CIT	Citrinin
CKD	Chronic Kidney Disease
CMIA	Chemiluminescent Microparticle Immunoassay
Cyr61	Cysteine-rich angiogenic inducer 61
DON	Deoxynivalenol
eGFR	Estimated Glomerular Filtration Rate
ELISA	Enzyme-Linked Immunosorbent Assay
FB1	Fumonisin B1
FDA	Food and Drug Administration
GDP	Gross Domestic Product
Gpx1	Cellular Glutathione Peroxidase
Gpx4	Phospholipid Hydroperoxide Glutathione Peroxidase
GSH-Px	Glutathione Peroxidase
GST- α	Glutathione Transferase Alpha
GST- α/µ	Glutathione Transferase Alpha/mu
H-FABP	Heart-type Fatty Acid Binding Protein
HGF	Hepatocyte Growth Factor
HK-2 cells	Human kidney 2 a proximal tubular cell (PTC) line derived from normal kidney
IARC	International Agency for Research on Cancer
IGFB-7	Insulin-like Growth Factor-Binding Protein 7
IL-10	Interleukin 10
IL-18	Interleukin 18
IL-6	Interleukin 6
KIM-1	Kidney Injury Molecule-1
L-FABP	Liver-type Fatty Acid-Binding Protein
LC3	Microtubule-associated proteins 1A/1B light chain 3B
*LCN2*	Lipocalin-2 gene (human)
*Lcn2*	Lipocalin-2 gene (rat, mouse)
MAP	Microtubule-Associated Proteins
MCP-1	Monocyte Chemoattractant Protein-1
MDA	Malondialdehyde
MHC	Major Histocompatibility Complex
MIF	Macrophage Migration Inhibitory Factor
mTORC1	Mechanistic Target of Rapamycin (mTOR) Complex 1
mTORC2	Mechanistic Target of Rapamycin (mTOR) Complex 2
NAG	N-acetyl-β-D-glucosaminidase
NGAL	Neutrophil Gelatinase Associated Lipocalin
NHE-3	Sodium–Hydrogen Exchanger 3
OAT	Organic Anion Transporters
OTA	Ochratoxin A
PAT	Patulin
PMTDI	Provisional Maximum Tolerable Daily Intake
PTWI	Provisional Tolerable Weekly Intake
RASFF	Rapid Alert System for Food and Feed
RBP	Retinol-Binding Protein
ROS	Reactive Oxygen Species
RPA-1	Replication Protein A1
sCr	Serum Creatinine
STC	Sterigmatocystin
TFF-3	Intestinal Trefoil Factor 3
TGF-β_1_	Transforming Growth Factor beta 1
Timp-1	Tissue Inhibitor of Metalloproteinase1
VEGF	Vascular Endothelial Growth Factor
WHO	World Health Organization
ZEN	Zearalenone
α-SMA	Smooth Muscle Alpha-Actin
